# Supplementary material for: An open-source framework for large-scale, flexible evaluation of biomedical text mining systems
Source: J Biomed Discov Collab. 2008 Jan 29;3:1. doi: 10.1186/1747-5333-3-1 (PMC2276192; doi:10.1186/1747-5333-3-1)
Supplement: Additional file 1 — Evaluation platform source code. The additional file contains the software evaluation framework discussed in this paper. The most current release can be downloaded from SourceForge.net [1]. See the accompanying README file for instructions on installation and use. Also included in the distribution is a collection of UIMA wrappers for some commonly used BioNLP tools and annotated corpora. [file 1747-5333-3-1-S1.zip › bionlp_uima_repository-1.0.1/docs/api/src-html/edu/uchsc/ccp/util/nlp/mention/ComplexSlotMention.html]

```
001    /*
002     *
003     * The Original Code is UCHSC Utilities.
004     *
005     * The Initial Developer of the Original Code is University of Colorado.  
006     * Copyright (C) 2005.  All Rights Reserved.
007     *
008     * UCHSC Utilities was developed by the Center for Computational Pharmacology
009     * (http://compbio.uchsc.edu) at the University of Colorado Health 
010     *  Sciences Center School of Medicine with support from the National 
011     *  Library of Medicine.  
012     *
013     *
014     * Contributor(s):
015     *   William A. Baumgartner <william.baumgartner@uchsc.edu> (Original Author)
016     *   
017     * Created: November 2005
018     * Dependencies:
019     * TODO: 
020     * Changes:
021     * 04/30/06 - wab - added getSingleLineRepresentation()
022     */
023    
024    package edu.uchsc.ccp.util.nlp.mention;
025    
026    import java.io.PrintStream;
027    import java.util.ArrayList;
028    import java.util.List;
029    
030    import edu.uchsc.ccp.util.nlp.annotation.TextAnnotation;
031    
032    public class ComplexSlotMention extends Mention {
033    
034        private List<ClassMention> classMentions;
035    
036        @Deprecated
037        public ComplexSlotMention() {
038            super();
039            classMentions = new ArrayList<ClassMention>();
040        }
041    
042        public ComplexSlotMention(String mentionName) {
043            super(mentionName);
044            classMentions = new ArrayList<ClassMention>();
045        }
046    
047        /**
048         * Get the class mentions that fill this complex slot
049         * 
050         * @return
051         */
052        public List<ClassMention> getClassMentions() {
053            return classMentions;
054        }
055    
056        /**
057         * Set the class mentions that fill this complex slot
058         * 
059         * @param classMentions
060         */
061        public void setClassMentions(List<ClassMention> classMentions) {
062            this.classMentions = classMentions;
063        }
064    
065        /**
066         * Add a class mention as a slot filler for this complex slot
067         * 
068         * @param cm
069         */
070        public void addClassMention(ClassMention cm) {
071            this.classMentions.add(cm);
072        }
073    
074        /**
075         * Return a string representation of this ComplexSlotMention in a single line.<br>
076         * Representation: " [mentionName]: [single-line-rep of ta linked to class mention]"
077         * 
078         * @return
079         */
080        public String getSingleLineRepresentation() {
081            String repStr = " [" + getMentionName() + "]:";
082            for (ClassMention cm : getClassMentions()) {
083                // repStr += cm.getSingleLineRepresentation() + ":";
084                TextAnnotation ta = cm.getTextAnnotations().get(0);
085                repStr += ta.getSingleLineRepresentation(true, true);
086            }
087            // repStr = repStr.substring(0,repStr.length()-1);
088            return repStr;
089        }
090    
091        
092        public String getDocumentLevelSingleLineRepresentation() {
093            String repStr = " [" + getMentionName() + "]:";
094            for (ClassMention cm : getClassMentions()) {
095                TextAnnotation ta = cm.getTextAnnotations().get(0);
096                repStr += ta.getDocumentLevelSingleLineRepresentation(true);
097            }
098            return repStr;
099        }
100        
101        public static void printMention(ComplexSlotMention csm, PrintStream ps, int indentLevel) {
102            String indentSpace = "-";
103            for (int i = 0; i < indentLevel * 4; i++) {
104                indentSpace += " ";
105            }
106            ps.println(indentSpace + "COMPLEX SLOT MENTION: " + csm.getMentionName());
107            indentLevel++;
108            for (ClassMention cm : csm.getClassMentions()) {
109                ClassMention.printMention(cm, ps, indentLevel);
110            }
111        }
112    
113        public String getHashKey() {
114            String key = this.getMentionName();
115            for (ClassMention cm : classMentions) {
116                key += (" " + cm.getHashKey());
117            }
118            return key;
119        }
120    
121        public int compareTo(Object o) {
122            if (o instanceof ComplexSlotMention) {
123                ComplexSlotMention compareCSM = (ComplexSlotMention) o;
124                return getSingleLineRepresentation().compareTo(compareCSM.getSingleLineRepresentation());
125            } else {
126                System.err.println("Unexpected object when comparing to ComplexSlotMention: object = " + o.getClass().getName());
127                return -1;
128            }
129        }
130    
131        
132        
133        @Override
134        /* Two complex slot mentions are equal if they have the same mention name, and their class mentions are also equal */
135        public boolean equals(Object obj) {
136            if (obj instanceof ComplexSlotMention) {
137                ComplexSlotMention csm = (ComplexSlotMention) obj;
138                if (this.getClassMentions().size() == csm.getClassMentions().size()) {
139                    /* if the cms's do not have an equal number of class mentions then return false */
140                    if (this.getMentionName().toLowerCase().equals(csm.getMentionName().toLowerCase())) {
141                        for (ClassMention cm : this.getClassMentions()) {
142                            boolean cmHasMatch = false;
143                            for (ClassMention cmToCompare : csm.getClassMentions()) {
144                                if (cm.equals(cmToCompare)) {
145                                    cmHasMatch = true;
146                                }
147                            }
148                            if (!cmHasMatch) {
149                                return false;
150                            }
151                        }
152                        /* if we get to this point, then all class mentions have a match, so return true */
153                        return true;
154                    } else {
155                        /* the csm's have different mention names */
156                        return false;
157                    }
158                } else {
159                    /* the csm's have an unequal number of class mentions */
160                    return false;
161                }
162            } else {
163                System.err.println("Cannot directly compare a ComplexSlotMention to " + obj.getClass().getName());
164                return false;
165            }
166    
167        }
168    
169        @Override
170        public int hashCode() {
171            return this.getSingleLineRepresentation().hashCode();
172        }
173    }
```
